# Using the Food Stress Index for Emergency Food Assistance: An Australian Case Series Analysis during the COVID-19 Pandemic and Natural Disasters

**DOI:** 10.3390/ijerph18136960

**Published:** 2021-06-29

**Authors:** Christina Mary Pollard, Timothy John Landrigan, Jennie Margaret Gray, Lockie McDonald, Helen Creed, Sue Booth

**Affiliations:** 1 Faculty of Health Sciences, School of Population Health, Curtin University, Bentley, WA 6102, Australia; timothy.landrigan@postgrad.curtin.edu.au; 2 East Metropolitan Health Service, Perth, WA 6000, Australia; 3 Western Australian Council of Social Service, East Victoria Park, WA 6101, Australia; jennie@wacoss.org.au (J.M.G.); helsak@westnet.com.au (H.C.); 4 Independent Consultant, Fullsky Consulting, Mount Lawley, WA 6050, Australia; lockie@fullsky.com.au; 5 College of Medicine and Public Health, Flinders University, Adelaide, SA 5000, Australia; sue.booth@flinders.edu.au

**Keywords:** food insecurity, disaster management, COVID-19, emergency food assistance, Food Stress Index

## Abstract

Food insecurity increases with human and natural disasters. Two tools were developed to assist effective food relief in Western Australia: the Food Stress Index (similar to rental stress, predicts the likelihood of household food insecurity by geographic location) and a basic and nutritious Food Basket Recommendation (that quantifies the types and amounts of food to meet dietary recommendations for different family types). This study aims to understand and compare the processes and impact of using these tools for organisations and their clients involved in emergency food assistance and/or disaster preparedness. A multiple case-study design analysed organisation’s use of the tools to assist the response to COVID-19 pandemic restrictions and the catastrophic bushfires in Australia. Qualitative interviews were conducted by telephone and Zoom (a cloud-based video conferencing service) in July–August 2020. A purposeful sample of eight interviewees representing seven cases (government, food relief and community organisations involved in emergency food assistance and/or disaster preparedness). Three themes emerged from the analysis, (1) organisations are confident users of the tools; (2) Collaborations were “*Ready to Go*” and (3) Food Stress Index is a “*game changer*”. Findings demonstrate the intrinsic value of the tools in the provision of emergency food relief under both normal circumstances and in times of increased need, i.e., COVID-19 pandemic. The study highlights the value and importance of ongoing intersectoral collaborations for food relief and food security (e.g., the Western Australian Food Relief Framework) and suggests that upscaling of the Food Stress Index and food baskets will increase the effectiveness of measures to address food insecurity in Australia.

## 1. Introduction

The population prevalence of food insecurity, when a household cannot access safe, nutritious, appropriate and affordable food to meet the families’ needs in socially acceptable ways, is not regularly or consistently measured in Australia [1]. Services supporting food-insecure families are interested in any information that can assist them in targeting and tailoring their assistance. Food stress, a similar concept to housing stress, occurs when a household needs to spend more than 25% of their disposable income on food [2]. Households most at risk of food stress are vulnerable to food insecurity because of inadequate income and factors related to financial hardship [3]. The Food Stress Index (FSI) was developed to provide a simple indication of the potential for food stress of households in a particular geographic location, e.g., for households in a Local Government Area [4]. It is a single index that encompasses socio-demographic, food costs and other determinants of food insecurity to provide information about the likelihood that households in a geographical area are suffering food stress. Specifically, the FSI’s multi-dimensional framework consists of household demographics, household income, household expenses, financial stress indicators, food security, food affordability and food availability. See the protocol paper for further information and details on the development of the FSI [4].

In 2020, a number of disasters, including bushfires, cyclones, storms and floods, as well as the COVID-19 pandemic food supply issues due to lockdown measures [5], led to the application of the FSI and related nutritious food basket recommendations. As well, the Western Australian Food Relief Framework Working Group (WAFR WG), a cross-sector collaboration originally brought together in 2015 to address the increasing demand for food relief, created a clearing house to coordinate sectors to target food relief efforts to areas in need.

The unique context of COVID-19 amplified the risk of food insecurity for those already at risk and created new demographics of food stress [6,7,8,9,10,11,12]. The economic impacts of COVID-19 and social isolation measures resulted in novel cohorts of people that suddenly found themselves at risk of food stress and food insecurity [13]. Simultaneously, the introduction of additional social protection payments, including supporting workers with JobKeeper (The JobKeeper Payment scheme was a subsidy for businesses significantly affected by COVID-19) and JobSeeker (The JobSeeker Payment replaced unemployment payments for Australians between 22 years and the Age Pension age and looking for work when they stopped in March 2020) allowances, and free childcare, youth allowance and parenting payments, meant that many of the cohorts usually living in poverty were protected [14,15].

Even prior to COVID-19, research highlighted that exposure to financial stressors, including the inability to get work, involuntary loss of work and inadequacy of social assistance payments, significantly increased the likelihood of food insecurity in Australia [16,17,18]. The Coronavirus Supplement payment doubled the rate of the JobSeeker payment during the pandemic and many of the groups that the food relief sector is more accustomed to working with no longer needed regular support. Subsequently, food insecurity in these groups reduced as they were able to afford food and other basic living costs. However, some new cohorts emerged, including international students [19,20] temporary visa holders, as they were ineligible for government support [12] and people impacted by natural disasters.

This is also substantiated by the Western Australia Council of Social Service’s (WACOSS) 2020 Cost of Living Report, where for the first time since analysis began, “*the unemployed single model household…exceeded their basic living costs*”. “The significant impact of extra financial support provided toward the end of 2019/20 financial year can clearly be observed to enable them to cover the cost of living essentials in line with community expectations” (pg 4) [21].

Instead, the sector’s strategic efforts were directed to those newly impacted groups, as well as communities whose access to safe and affordable food was considerably curtailed due to travel restrictions. During the height of the COVID-19 lockdown in 2020, WAFR WG met weekly. Representatives of this group used the FSI and the food basket recommendations to identify areas of need and where to direct food relief efforts, including the quantum of food required.

The aim of this study was to get a more in-depth understanding of each organisation’s use of the Food Stress Index and food basket recommendation tools overall, with a particular focus on food relief during COVID-19. Our hypothesis is that the Food Stress Index, when used in conjunction with food basket recommendations, is an effective tool for targeted food relief efforts.

## 2. Methods

### 2.1. Study Design

A comparative multiple-case design of organizations who have used the FSI and/or food basket recommendation tools, particularly during the COVID-19 pandemic in Australia. Case studies were selected as they can explain, describe or explore events or phenomena in the everyday contexts in which they occur. They can help to understand and explain the links and pathways resulting from a new policy initiative or service development.

A comparative case study design was chosen for four main reasons. Firstly, the approach is suitable in examining when how and why questions are being posed about the processes or outcomes of an intervention. Secondly, they are useful in understanding and explaining how context influences the success or failure of an intervention. Thirdly, they are feasible in contexts where an experimental design is not possible, and finally, comparative case studies often use both quantitative and qualitative data.

In this study, one or more interventions (the application of the FSI and/or the food basket recommendation tool) are being implemented across multiple contexts, and there is little opportunity to control the way in which the tools are being used. As such, the context in which the tools are being used is very important in understanding the success or otherwise of the intervention. This study was approved by the Curtin University Research Ethics Committee Approval Number HREC 2020-0326.

### 2.2. Case Definition

The case of interest in this study centred on the detailed description and practical application of the FSI and food baskets recommendation tool for food relief/disaster management scenarios. The selection of the cases is based on activities that merit some reflection. These case studies have been carefully selected to represent a range of responses to the application of the tools and the work of the WAFR WG.

### 2.3. Recruitment

A purposive sampling technique identified cases for inclusion with the key criteria exploring scenarios where Australian organisations involved in emergency food assistance had used the FSI. Organisations and potential interviewees were identified by members of the WAFR WG. The members had contact with organisations involved in food assistance and knew who had used the tools in the previous six months.

Fifteen cases were initially identified, and following an email approach letter from the lead author (C.M.P.), seven organisations were willing to be interviewed. Organisations were excluded if they had not used the FSI or if they did not respond to two emails and one follow-up telephone call. See Table 1 outlining cases.

### 2.4. Data Collection

Interviews were conducted by Sue Booth (S.B.), a qualitative researcher who was not a member of the WAFRF W.G. Prior to interviews, S.B. was provided with a brief precis of each of the seven cases. These were drafted by the remaining authors, who were all members of the WAFRF W.G. C.M.P. and S.B. then used this information to inform the framing of interview questions.

Interviewees were contacted by email to schedule the interview and provided with a Study Information Sheet. Consenting interviewees participated in an interview lasting between 40–60 min. The interviews were conducted by telephone and videotelephony (Zoom) between July and August 2020 as this method was appropriate during COVID-19 physical distancing measures [22] and audio-recorded on an Olympus WS-832 digital voice recorder. All interview audio recordings were transcribed verbatim by a professional digital audio transcription service.

A semi-structured interview instrument (developed by C.M.P. and S.B., discussed and edited by Lockie McDonald (L.M.), Jennie Gray (J.M.G.) and Helen Creed (H.C.)) explored: the participant’s understanding of the FSI and food basket recommendations; the circumstances that lead to their use; use of the tools during the COVID-19 pandemic; on how well the tools performed during the event; and other uses of the tools.

### 2.5. Data Analysis

A two-stage analysis process was undertaken to understand (i) how the FSI and food basket recommendations had been applied and (ii) examine what worked in different contexts across the seven cases. Firstly, a systematic comparative case analysis was undertaken to describe the context, compare the key dimensions and anticipated outcomes. After transcription, all case study data was thoroughly read (S.B.), and each individual case study was summarised. Constructing the case summaries was a way to familiarise the researchers with each case and limit the possibility of inaccurate coding. Prior to commencing coding, the case summaries were provided to the research team, who read, discussed and recorded their initial impressions of the cases.

The interview data and case summaries were entered into NVivo, where they were carefully read through several times to build familiarity with the data. Open and axial coding of the data was undertaken. A list of nodes based on repetitive patterns and themes found throughout the cases was developed. Data coded at nodes were reviewed and discussed (S.B. and C.M.P.). Overlapping codes were collapsed, and emergent themes were noted.

**Figure 1:**
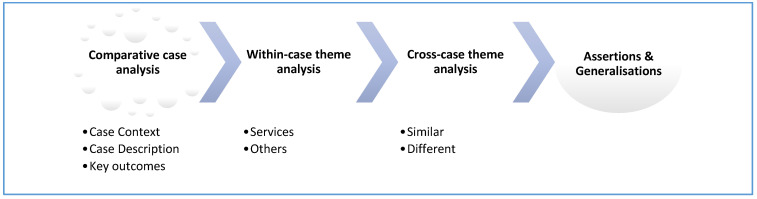
The process in the comparative cases study analysis (adapted from ten Bensel and Sample (2015) [23]).

The second stage involved explanation building, i.e., the goal is to analyse case study data by building an explanation regarding the uptake, process and sequence of tool use across the cases. Data analysis in this stage used case comparison diagrams within NVivo to explore and build an explanation. See Figure 1.

## 3. Results

All cases used the FSI, and four used the food basket recommendations. Seven organisations were represented, including Emergency Food Relief providers and/or direct services (*n* = 3), advocacy organisations (*n* = 2) (local government and community service sectors), a remote health service and the Federal Government Department (Social Services).

### 3.1. Case Context and Description

Seven specific scenarios or events where the FSI was applied represent the cases. Table 1 provides a brief description of each case. The case studies focus on the use of the FSI that highlighted geographic areas at greatest risk of food stress and nutritious food basket recommendations developed to meet the nutrition requirements of different family group scenarios. The practical baskets identified the types and amounts of food for all nutrient requirements and included recipes and menu plans. Combined with the information from the FSI, the specific family composition by postcode, the quantum of food (e.g., the types and amounts for each family for a week by any geographic area) could be estimated. Areas of focus during the COVID-19 were identified using the FSI and represented existing low-income communities, hard hit by COVID-19-related job losses. Case 4 includes the use of materials for the catastrophic Australian bushfires in Victoria in January 2019.

### 3.2. Within Case Themes

**Figure 2:**
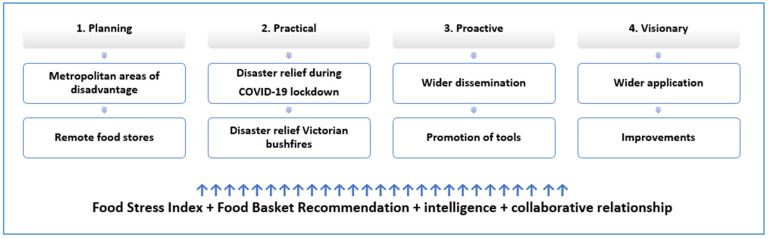
The stages of the process of how the Food Stress Index and food basket recommendations were applied by emergency food relief providers.

The emergency food relief providers (Cases 1, 4 and 6) described a process and patterns of use of the FSI and food basket recommendations that were consistent across cases (Figure 1). Furthermore, the tools were used in a similar way before and during the COVID-19 pandemic. These cases highlighted four stages of application of the FSI: Planning (stage 1); Practical (stage 2); Proactive (stage 3); and Visionary (stage 4), see Figure 2.

Stage 1: During the Planning Stage, the FSI and the food basket recommendations were used as a cross-check validation measure in conjunction with other information (e.g., local intelligence, local government data) to determine the extent and geographic location of food need. During the COVID-19 pandemic, service providers could predict “hots spots” and areas of high need as described here:

“*We were cross-checking the food index tool and the map that was developed out of the tool, into our areas of need around the State. [name] is probably the largest emergency relief program in WA… we have a really good snapshot because we service around 50,000 to 55,000 clients a year… So, with the food index tool, we started to look at that and to map out our emergency relief data and our financial counselling data, and then that helped to determine our hot spots around what we thought would happen with food for our planning for this year*.”

(Case 6)

Stage 2: During the Practical Stage, the FSI and the Food Basket Recommendation were used to confirm the location and predict the “quantum” of food to be delivered. Critical to the operation’s success was a range of collaborative relationships to assist in the food relief operation. These included food donations or purchases from major supermarket chains, freight logistics from trucking companies, staffing/on-the-ground personnel from local governments and the use of storage space and refrigeration from community clubs and services. Existing relationships and their extensive networks (including those who were not usually emergency response providers) assisted organisations to “pivot” to respond. For example, community resource centres (CRCs), of which there are 100 organisations in regional areas [24], assisted in distributing sufficient food to communities in a timely manner.

“*We saw we had all the tools and predictions there, probably within the second week of April. We had everything predicted and worked out really quickly. Then it became a logistical thing because, if we were all in closedown, you didn’t have access to logistics of getting food into the State. So, then that then became the headache. The actual planning was good, and I had made a couple of large food orders before COVID broke and we went into lockdown, so we had a good chunk of food in stock pre-lockdown. And then probably five weeks into COVID, probably mid-April, same thing in that period. We managed to convince Coles and IGA and Woolworths to open up their orders to us on a larger scale*….”

(Case 6)

Stage 3: During the Proactive stage, buoyed by the success and confidence of using an evidence-based tool to respond to a food need, Emergency Food Relief providers were actively promoting and widely disseminating the tool/s to colleagues both within the State and in other jurisdictions.

“*I have to also say I have pedalled the Food Relief—the Food Stress Index and the Healthy Food Basket in every other state in the country as the exemplar of the kind of information you need to build the responses that they’ve developed in each of those states. So I am, around the rest of the country, actually the number 1 salesman*.”

(Case 4)

Stage 4: During the Visionary Stage, having successfully used the tool/s, Emergency Food Relief service providers were cognisant of the value of the tool/s; specifically, the wider application, expansion, and possible improvements for both tools. For example, including multi-layered demographic data related to poverty such as income, social assistance, housing data to inform poverty reduction strategies and social policy responses.

“…*My contention is that the whole development of both of these instruments is not only the bedrock for resolving food relief, it is the potential keystone information for resolving a whole series of unrelated social issues, in terms of the amount of things that food relief and poverty inform. So, I think it’s a predictor of health spend, I think it’s a predictor of social work spend. I think it’s a predictor of—there are so many things you can link to that granular understanding of poverty generally and how it impacts your entire population. But I think it has a use to predict the value in a whole series of arenas that are not currently being considered*…”

(Case 4)

“*I think that the Food Stress Index should be applied by government, not only state but Federal. Looking at resourcing areas, looking at what’s available in those communities and also not just around food, but also other resources, such as infrastructure. Because …the Food Stress Index actually just reflects poverty in Australia per se, if you look at all the composite measures that are going into it as well. So it’s a barometer, … and … the government and other organisations should be using the Food Stress Index for where they’re trying to provide support to low income people….., let’s look at this area as even if we’re looking at payments for welfare, we can identify that here, there’s higher need. And okay, so what else do we need in addition to welfare? Well, we also need food, … infrastructure. And we need education. We need support. We need mental health. All those sorts of things that go and wrap around with people who are experiencing food insecurity or food stress*.”

(Case 1)

Other cases described the value of the tools in similar planning, practical, proactive and visionary ways but, as a result of their organisational role, did not demonstrate a systematic use across all four stages. For example, the Federal Government Community Service case study used the FSI for planning, i.e., data cross-checking with other intelligence sources and the Social Services advocacy organisation, does not provide food relief (stage 2 practical) but applied the tools in all other stages. Cases showed that the benefit and future application for alleviating poverty of the tools were evident in organisations that did not use the tool in practice.

### 3.3. Cross-Case Themes

Three themes emerged across all cases regarding the use of the Food Stress Index to guide food assistance for disaster management: (i) Adopters are confident users; (ii) Collaborations were “*Ready to go*”; (iii) the Food Stress Index is “*a game changer*”.

(i) Adopters are confident users

Cases showed how the new FSI and the food basket recommendation tools could be used to deliver emergency food relief in response to poverty or an unforeseen disaster. They made sense to all those who had seen them, and respondents described with confidence how they had applied them. The food relief sector applied them and improve their service delivery, as indicated by the quote below:

“… *And so right from the first point, where this was part of the conversation, it immediately dawned on me that this data [provided by the Food Stress Index and Food Basket tool] was the key to understanding what we needed to do in instances of total relief, but also in instances of partial relief and what the extent of that might look like and where it would be located. So, it was like meeting someone who had the key to Aladdin’s Cave*…”

(Case 4)

The relief sector were keen supporters of the evidence-based practice tools to guide their work and felt a sense of relief and gratitude that they were now available. They reflected that the tools facilitated an operational shift away from “ad hoc” to “evidence based” actions. Once they used the tools, they saw their value in practice and started promulgating and recommending them. Emergency food relief providers and peak advocacy bodies, as well as government funders of social services, described their support and enthusiasm for the tools, as shown in the quotes below, firstly emergency providers:

“… *I would say most definitely the Food Stress Index continually provides a reference for myself and my organisation to go, absolutely look at this is a population or a community in high need. There’s no question around providing food or resources to that area*…”

(Case 1)

“*Everybody you present this to [tools] say it’s—almost uniformly, the response is thank Christ, we’ve finally got some good information to work from*.”

(Case 4)

Secondly, local government peak,

“*We used it perfectly, I believe, during the COVID response, just in the way I outlined by using it as a formalised policy and procedural framework that was backed by a robust research and dataset. So, we were able to use that without a doubt*.”

(Case 5)

and the health service,

“… *I’d plenty of other work to do so I didn’t have a lot of time to sit down and work it out, but it was already done, and I could see that it worked*…”

(Case 2)

(ii) Collaborations were “*Ready to Go*”

Cases illustrated that strong partnerships between and across sectors were critical to being able to deliver a quantum of food to communities in need. Whilst it was the tools that helped to identify the area of need and the quantum of food, it was the relationships with partners and collaborators that made it all happen. Collaborators included other emergency food relief providers, major supermarket chains, local government, mining companies, philanthropists, freight logistics and trucking companies, and local communities, as illustrated in the quotes below:

“*The Freight and Logistics Council of WA…. was fundamental. [name blinded] through her roles in the council as well as Infrastructure WA has very well established and respected relationships with a lot of the food suppliers across the state. And a deep understanding of the logistics including storage and distribution…. So, she used her relationship with Metcash to just get an instant allocation of food and in accordance to the basket and then the delivery of it down to a site, what they didn’t have there was storage. So that’s when the shire stepped in and they were able to provide a facility that had ingress/egress according to COVID requirements and social distancing requirements as well as dry and cold storage facilities on site*.”

(Case 5)

“*Oh, during the first seven weeks of the COVID lockdown period in the first round, [A major supermarket chain] donated half a million dollars’ worth of product to us on a weekly basis for that seven-week period. So, they are a real national partner and they’re a very engaged partner. And even in the procurement process they offered us truly significant discounts, with respect to the—and we were spending federal money, so we needed to derive value from it. But they made sure we got better value than we got anywhere else, better choice, better selection and delivery as required*.”

(Case 4)

The case descriptions suggest collaborators mobilized rapidly during the COVID 19 pandemic and that individuals and agencies went to additional efforts to ensure food was available for distribution. “*Crates of food would just turn up*” donated from major supermarket chains, and food would be delivered to bushfire affected communities and the bill for trucking absorbed by the freight company. Unusually, food was provided upfront and “in good faith,” with invoices for payment to follow. Mining “*companies all came together to make a donation, which then provided for hampers to go to the remote communities*”.

The quote below demonstrated how the different sectors worked together quickly,

“… *You have got to have them [tools plus resources, logistics] all working in conjunction. There’s no point putting a recommendation through to the department if there’s a cyclone or something and then nothing happens for four weeks, because the need is there instantly, and you have to move instantly*.”

(Case 6)

Historically competitive relationships regarding food procurement arrangements between emergency food providers were put aside during the pandemic in favour of genuine collaborations to share food items with other providers to complete hampers for distribution.

“*We had Second Bite, which is another food provider that was also providing some fresh fruit and veg for a while, and they were part of that as well. Yeah. It was amazing…, it was such a proactive food relief program. It was extraordinary…, the amount of clients and people we supported, and are still supporting, has been amazing*.”

(Case 6)

The cases highlighted the benefit of the WAFRF WG set up as a clearing house mechanism for ongoing relationships, co-operation and intelligence sharing and how this had contributed to the timely co-ordination and delivery of emergency food relief. The WAFRF WG allowed for intelligence sharing amongst emergency relief sector members with respect to areas of need, food donations available, resourcing, storage and transport logistics. The quotes below are typical:

“… *by having the framework already in place and established … pre-COVID, there was already a set understanding and sets of definition of what the basic human needs are around food. So, a lot of those questions had already been answered; a lot of those stakeholder relationships had already been pulled together …The early work had already happened through those conversations….it wasn’t a question of should we or shouldn’t we. It was yes, this is something that we will do because all of that work’s already been done*…”

(Case 5)

“*So I think if we didn’t have the Food Relief Framework, we would have seen countless kilograms of food being ploughed into the ground or waste, it was only because of that interconnectivity across government, across not-for-profits and industry that we were able to have that food redirected to the community and to where it was needed. So, I think it is unique … [If] you don’t have a Food Relief Framework in the rest of the country … it just seemed to be a chaotic donation system and delivery and dissemination of food system. … And not all players at the table. Whereas the framework has all the players at the table. And everybody’s represented. Particularly, the people in need*.”

(Case 1)

(iii) The Food Stress Index is a “game changer”

All cases highlighted the importance of the FSI and the food basket recommendations in the delivery of food relief, describing them as *“a bedrock piece*” and a *“real golden nugget”* in responding to community needs. The use of the tools allowed for a sensitive, tailored response, as illustrated below:

“*But if we hadn’t had the Healthy Eating Basket and the experience of working in Western Australia, we wouldn’t have been able to compose on or bring the bulk supply in totality to try and get it across a weekend. And without (1) the experience of doing this and understanding it in Western Australia, and (2) without having had the Healthy Eating Basket, we would have been guessing. We would just have been trying to pool together whatever the hell we could in a short period of time and ship it out and just hope that the other components would come … [it], allowed us to response in a way that we’ve never been able to respond before. It was, in some respects, our finest hour, because we fit the job more comprehensively and to a better standard, both in terms of quality and nutrition, than we have ever been able to at any point in our history*.”

(Case 4)

“*They are both excellent tools and, from Vinnies, the impact we’ve had by the use of those tools has been incredible. Just the numbers of people, the comments that we’ve got back from agencies around the pressures that it’s taken off families*.”

(Case 6)

Emergency food relief providers, in particular, noted the tools were critical in driving a shift in the way their operations were conducted, i.e., from anecdotal to evidence-based practice. In this way, the “talk” suggests the tools have the potential to *“change things across the country*” with respect to emergency food relief provision. The quotes below were typical:

“… *we’re always wanting to use evidence-based tools and I think it’s a really informed tool and it makes it a practical tool*.”

(Case 2)

“*It’s changed the way we understand food insecurity. It’s changed the way we think about responding to food insecurity. And it’s changed the way we think about providing food. It’s just changed everything. Before the Food Stress Index, … I really didn’t understand food stress and insecurity.., and I don’t think many of the providers did either. Now they understand, they understand what food stress is, they understand it’s a pre-step to food insecurity. They understand more clearly that people experiencing food stress and insecurity need an appropriate response across nutrition, across chronicity. Yeah, I think it’s just changed everything*.”

(Case 7)

The tool’s wider applicability to address other social policy issues underpinned by poverty was highlighted by several cases. Use of the tools for planning resources allocation, funding, advocacy and strategic policy was noted along with expansion to include the overlay of other relevant poverty-related data such as housing and mental health. Federal level use of an expanded tool could inform policy and funding decisions. The quotes demonstrate their potential applicability,

“… *there’s also an advocacy and policy piece. So, what we could do with the Food Stress Index is we can: (a) be able to identify the movement and changes of food stress across different suburbs, and then use that data in addition to all the other data we use for our advocacy and policy work with government. And then we would be able to use the Food Stress Index as another piece of evidence*.”

(Case 7)

“… *In everything. A bit of Food Basket, Food Stress Index and the framework. I see that playing a key role in everything we do in social welfare or social policy, because if we go back to Maslow’s Hierarchy of Needs, everyone has the basic right to food and accommodation, fresh water, food and accommodation. So, without somewhere to live and something to eat, everything else falls apart. So, if we get those two things right first then we can start to build on a more empowered and actualised life*.”

(Case 6)

The assertions and generalisations drawn from this analysis are outlined in the Discussion.

## 4. Discussion

This study has described the use and application of the novel FSI and food basket recommendation tools amongst multiple emergency food relief cases. A consistent pattern of use emerged across cases which consisted of four stages: planning, practical, proactive and visionary, suggesting high levels of applicability of the tools across scenarios.

These case studies revealed how the emergency food relief sector had little evidence, if any, to rely on to estimate the level of need (demand) and the types and amounts of food required (supply) to plan their responses to communities in need. Recent natural disasters and the COVID-19 pandemic highlighted limitations within the current food relief system, particularly the fragility of food logistic supply chains. The case studies also revealed how the lockdown measures due to COVID-19 meant that services had to “pivot” to contactless systems, e.g., from volunteers supplying meals to prepacking hampers for distribution through alternative channels. Importantly, case studies suggested that services were already aware of the shortcomings of their “usual ways of doing business” and were seeking urgent solutions in response to a series of increasing, cascading and concurrent disasters in Australia.

The WA Food Relief Framework was perceived as a critical mechanism that enabled timely and effective collaboration with others in the pursuit of more streamlined and effective service delivery. Interviewees recognized the value of established networks and collaborations within the food assistance sector during times of crisis. It was these relationships and goodwill that saw “competitors” (organizations within the same sector who compete for funding) work together to deliver food supplies to where they were needed. There was a general agreement that without the high level of intra-sectoral trust and co-operation that was established previously through the work of the Framework, the delivery of food relief would not have been as streamlined. The networks established a notion of shared responsibility and facilitated an integrated cross-sectoral systemic response to disasters and uncertainty. The whole-of-community response involved a range of actors, including the private sector, public sector and Third sector (e.g., non-government organizations, not-for-profits), as well as community leaders and volunteers. The emergency relief sector worked closely with the food industry, freight/trucking logistics and community groups on the ground to source and deliver food relief.

The studies revealed that food donations usually make up the majority of the food supply, and the food relief sector has limited control over the types, amounts or nutritional quality of food received. Notably, COVID-19 response measures provided funding for the food relief sector to purchase additional food. The interviewees were all aware of the need to provide culturally appropriate, nutritionally adequate food, in part due to their engagement with the WAFRF and based on their wealth of experience. These conditions may have contributed to the rapid uptake and use of the tools that were seen as ensuring nutritious food provision.

There is also no guidance on the types and amounts of food that should be provided to meet nutrition needs or how to provide food for populations requiring emergency assistance in Australia. The shortcomings of this lack of formal food and nutrition policy were reinforced and brought to stark public attention in Melbourne during the July 2020 sudden strict coronavirus lockdown of a public housing complex. During this time, over 3000 residents from diverse cultural backgrounds were unable to leave their apartments to purchase food for over a week. Food relief agencies were not able to deliver culturally or nutritionally appropriate food, causing significant concern about resident’s well-being and prompting public outrage [25]. There is an urgent need to develop agreed national culturally appropriate, nutritious food basket recommendations for emergency food relief.

There are also no formalized Australian food assistance guidelines for disaster management. The absence of National disaster food relief guidelines is becoming increasingly problematic as the frequency of climate change-induced natural disasters (e.g., floods, bushfires and cyclones) increases, along with COVID-19 related lockdowns. In both these examples, the use of the tools would have increased the capability of services to deliver the quantum of nutritious food required for families. Consistent with this, our study found that users of the FSI and recommended food baskets were able to see the wider applicability of the tool across different scenarios (Stage 4 Visionary), overlaid with additional data such as housing status to predict areas of need and address poverty more broadly.

**Figure 3:**
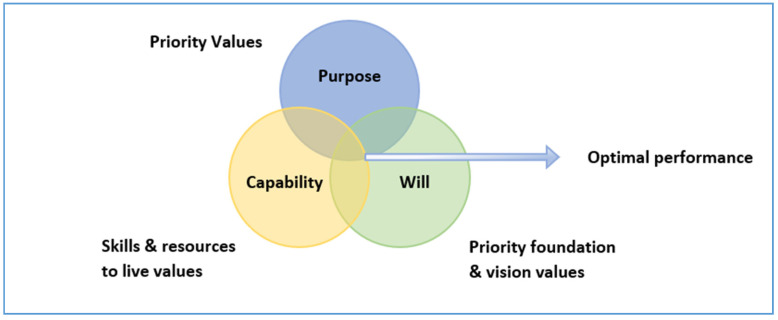
The Optimal performance model (adapted by Smith and Saint-Onge 1996, pg 16 [27]).

This case study research demonstrates that the FSI tool was effective in building the capability of a range of stakeholders. Values alignment is an important organizational property to guide a critical mass of people towards an organizational goal or mission [26]. The Optimal performance model (adapted by Smith and Saint-Onge 1996, pg 16 [27]) provides a useful framework for reviewing organizational-wide values and setting values alignment. See Figure 3.

Capability, purpose and will are values required to achieve optimal performance. Applying this framework to our study cases, the majority of cases had the purpose and will to achieve their organizational goals; however, the capability was lacking. The FSI tool provides the missing capability piece in this framework and along with existing purpose and will, leading to optimal performance in an emergency food relief/disaster relief context.

Furthermore, this study found high levels of collaboration across cases but especially those involved in emergency food assistance. Drawing on Figure 3, we argue that having the critical elements of values alignment (purpose, will and capability—in the form of the FSI) fosters better inter- and intra-sector collaboration.

The current study findings build on the research examining the disaster response to achieving resilience food systems following the extensive flooding in Queensland in 2011, which concluded that adaptive governance was necessary to ensure responsibility, participation and collaboration between local and other levels of response. [28]. The WAFRF WG and clearing house was critical in ensuring local coordination and an adaptive response.

In summary, as new cohorts of food insecure people emerged during the pandemic, the value of the tools and the WAFRF WG was evident. Firstly, the FSI had already highlighted and quantified the households in the geographic areas most likely to be at risk of food insecurity (the FSI value predicted the number of households in each geographical area at risk of food insecurity, and the baskets estimated the types and amounts of food needed to respond). Secondly, and most importantly, during the pandemic response, new cohorts of food insecure people were identified by services (e.g., international students who were not eligible for social assistance) and communicated through the already established WAFRF WG collaboration and the weekly clearing house meetings.

As a result of the case study series analysis examining the use of the FSI and food basket recommendations, we assert that these tools and the cross-sector collaborative framework established (i.e., the WAFRF) are consistent with the eight principles of emergency planning outlined in the Australian Institute of Disaster Resilience’s Emergency Planning Handbook (2020). Nationally agreed principles for good practice in emergency planning practice include: to be risk-informed, reduce unknowns, to be collaborative and inclusive, strategic, solution-oriented, iterative, to enable adaptive capacity and share responsibility [29]. The case studies demonstrate that when used collectively, the three components, (i) The WA Food Relief Framework, (ii) Food Stress Index and (iii) food basket recommendations, underpin best practices not only in emergency relief but also in disaster response.

## 5. Study Limitations

In interpreting the results from this study, it is important to recognise the limitations. The study was conducted in Western Australia to describe a small number of events and use of the FSI and food basket recommendations between June 2019 and July 2020, and specifically during the early stages of the COVID-19 pandemic and response to restrictions in Western Australia. This case study series involved a small number of organisations utilising the FSI and recommended food baskets for emergency food relief and disaster preparedness. It is recommended that the tools be made more widely available and developed further to be suited to specific scenarios, for example, recommended dry food baskets for packing 3 to 5 months in advance for disaster planning, with additional hybrid baskets for inclusion of perishable food when available for ongoing food relief, and vegetarian, pasta-based and rice-based baskets to better meet the needs of culturally diverse dietary patterns. As these tools proved useful in assisting services to source nutritious and appropriate food for families, it is recommended that the FSI and recommended food baskets on which is it based be made available Australia-wide to support national efforts. Despite these limitations, the potential for the application of the FSI in other countries. Men et al. (2021), when considering the association between household food insecurity and mortality in Canada, recommended that “*a composite measure like the Food Stress Index might better capture geographic contexts relevant to food security such as living costs*” pg7 [6].

A limitation of the current study is the focus of the application of the FSI on the series of cases conducted during 2020 in response to emergency food relief provision due to natural disasters, COVID-19 pandemic and related restrictions and disaster preparedness scenarios. As suggested by the interviewees, some of whom advocate to address the causes of food insecurity, these tools have wider application. In the absence of routinely collected household food security data, the FSI predicts the likelihood of food insecurity based on an index [4] created using statistical area one household characteristics from national census data, socio-demographic household characteristics associated with food insecurity in Western Australia [30] and the cost of a basic, nutritious food basket by geographic location [31]. The FSI can highlight areas of a high likelihood of requiring food relief; however, not all households who are food insecure require food relief as there are other coping strategies available. Within the Australian context, specific criteria contributing to the FSI value are amenable to change with Government intervention, e.g., social protections to increase minimum welfare payments, or minimum wage, or intervention to reduce casualisation of the workforce etc. The impact of some of these protections was seen at later stages of 2020 when recipients of government financial payments reported a reduced incidence of food insecurity and an ability to purchase food in dignified and socially acceptable ways [32].

## 6. Conclusions

The case study analysis has demonstrated the applicability and usefulness of the Food Stress Index and nutritious food basket recommendations in identifying and responding to episodes of food insecurity due to poverty, natural and other disasters. The findings showed a rapid uptake by the food relief sector direct services, the relevance of the tools and that they were easy to use. Results suggest that the tools built each organisation’s capacity to respond to a variety of food relief scenarios, including the unprecedented circumstances that presented during the COVID-19 pandemic and in response to lockdown measures and natural disasters. Findings described how the tools assisted services’ “business as usual” activities and improved their efficiency and effectiveness, supporting a more professional response. The significance of these tools in assisting food relief efforts is apparent in the current study. Overlaying mitigation activities such as government social assistance payments and other services onto the Food Stress Index could markedly improve responses to the complex determinants of food insecurity. With increasing and concurrent disasters, the time is right to make these tools routinely and freely available.

## Figures and Tables

**Table 1:** Brief case study organisational description, context and scenario.

Case	Type	Broad Aim	Area	WAFR	Event or Circumstances that Required the Use of Tools
1	Food bank (C)	Provide access to nutritious food assistance	WA *	Yes	Requested a FSI presentation, then anecdotal evidence suggested they used the FSI to identify areas for their pop-up food vans, and to identify priority geographical locations for food relief. They used the food basket recommendations by location to redirect and quantify food relief / hamper needs. COVID-19 isolation requirements meant volunteers and recipients could no longer attend the food bank, so pivoted to pre-packed and stored nutritious hampers with constraints one weight, perishability, and family composition.
2	Health service (C)	Provide health care services and programs in one remote area	Remote	No	Commission to support remote Indigenous communities with their food related disaster management planning during the COVID-19 Pandemic. Advice on pre-packed and stored nutritious hampers with constraints one weight, perishability, and family composition.
3	Social Services (G)	Improve the wellbeing of Australian families	WA *	No	Member of Fair Food, fund emergency relief services (includes food relief) based on grant submission process. Requested a FSI presentation to provide insights as part of their service review. Interested in sustained, positive community impact of funding.
4	Food rescue (C)	Food rescue network for redistribution to charities	National	Yes	Food rescue organisation that negotiated with major supermarket chains to procure donated food for collaborative food relief during natural and health disasters e.g., bushfires, floods, and the COVID-19 Pandemic. Used the FSI and basket recommendations to identify geographical areas for food relief and to estimate quantities of food needed.
5	Local Gov. peak body (NP)	Assist local Governments to meet community needs	WA *	Yes	Attended FSI presentation and used the FSI to share information with local governments with high likelihood of food stress. During the COVID-19 lockdowns and response shared data with all levels of government and were essential in assisting inter-sectoralresponses.
6	Lay catholic (C)	Shaping a just and compassionate society	WA *	Yes	COVID-19 lockdown caused a food assistance model pivot to achieve contactless delivery of food hampers (e.g., no volunteer delivery into homes). They moved to non-perishable food hampers prepared ahead of time, stored onsite, and distributed using a ‘drive through’ model. Food hamper contents were derived using the FSI food basket recommendations applied to a set weight, family composition, number of days, price, and storage capacity of three months.
7	Community Service peak body (NP)	Drive social change to ensure justice & equity	WA *	Yes	Advocacy organisation coordinating food relief services, central information sharing conduit, host and maintain online information sharing platform and service provider networks. Pivotal for government relationships regarding food during COVID-19 pandemic.

* Denotes WA branch of a national organisation; (C) = Charity; (G) = Government; (NP) = Not for Profit.

## Data Availability

Restrictions apply to the availability of these data. Data was obtained from participants and are confidential.
